# Responses to Pattern-Violating Visual Stimuli Evolve Differently Over Days in Somata and Distal Apical Dendrites

**DOI:** 10.1523/JNEUROSCI.1009-23.2023

**Published:** 2024-01-31

**Authors:** Colleen J. Gillon, Jason E. Pina, Jérôme A. Lecoq, Ruweida Ahmed, Yazan N. Billeh, Shiella Caldejon, Peter Groblewski, Timothy M. Henley, India Kato, Eric Lee, Jennifer Luviano, Kyla Mace, Chelsea Nayan, Thuyanh V. Nguyen, Kat North, Jed Perkins, Sam Seid, Matthew T. Valley, Ali Williford, Yoshua Bengio, Timothy P. Lillicrap, Blake A. Richards, Joel Zylberberg

**Affiliations:** ^1^Department of Biological Sciences, University of Toronto Scarborough, Toronto, Ontario, Canada; ^2^Department of Cell & Systems Biology, University of Toronto, Toronto, Ontario, Canada; ^3^Mila, Montréal, Québec, Canada; ^4^Department of Physics and Astronomy, York University, Toronto, Ontario, Canada; ^5^Centre for Vision Research, York University, Toronto, Ontario, Canada; ^6^Allen Institute, Seattle, Washington; ^7^Département d’informatique et de recherche opérationnelle, Université de Montréal, Montréal, Québec, Canada; ^8^Learning in Machines and Brains Program, Canadian Institute for Advanced Research, Toronto, Ontario, Canada; ^9^DeepMind, Inc., London, United Kingdom; ^10^Centre for Computation, Mathematics and Physics in the Life Sciences and Experimental Biology, University College London, London, United Kingdom; ^11^School of Computer Science, McGill University, Montréal, Québec, Canada; ^12^Department of Neurology & Neurosurgery, McGill University, Montréal, Québec, Canada; ^13^Vector Institute for Artificial Intelligence, Toronto, Ontario, Canada

**Keywords:** distal apical dendrites, hierarchy, neocortex, pyramidal neurons, sensory prediction, unsupervised learning

## Abstract

Scientists have long conjectured that the neocortex learns patterns in sensory data to generate top-down predictions of upcoming stimuli. In line with this conjecture, different responses to pattern-matching vs pattern-violating visual stimuli have been observed in both spiking and somatic calcium imaging data. However, it remains unknown whether these pattern-violation signals are different between the distal apical dendrites, which are heavily targeted by top-down signals, and the somata, where bottom-up information is primarily integrated. Furthermore, it is unknown how responses to pattern-violating stimuli evolve over time as an animal gains more experience with them. Here, we address these unanswered questions by analyzing responses of individual somata and dendritic branches of layer 2/3 and layer 5 pyramidal neurons tracked over multiple days in primary visual cortex of awake, behaving female and male mice. We use sequences of Gabor patches with patterns in their orientations to create pattern-matching and pattern-violating stimuli, and two-photon calcium imaging to record neuronal responses. Many neurons in both layers show large differences between their responses to pattern-matching and pattern-violating stimuli. Interestingly, these responses evolve in opposite directions in the somata and distal apical dendrites, with somata becoming less sensitive to pattern-violating stimuli and distal apical dendrites more sensitive. These differences between the somata and distal apical dendrites may be important for hierarchical computation of sensory predictions and learning, since these two compartments tend to receive bottom-up and top-down information, respectively.

## Significance Statement

Hierarchical predictive computation is believed to be a major function of the neocortex. However, it is unknown whether stimuli that violate previously-experienced sensory patterns induce different responses in the compartments of neurons where bottom-up and top-down signals are predominantly integrated. Here, we track the responses of different compartments of neurons in mouse visual cortex as we present animals with pattern-violating and pattern-matching visual stimuli. In the neuronal compartments that receive bottom-up and top-down signals, we find that the responses to pattern-violating compared to pattern-matching stimuli evolve differently over time. This may provide critical insight into hierarchical sensory computation and predictive learning in the brain.

## Introduction

A long-standing hypothesis in computational and systems neuroscience is that the neocortex learns a hierarchical, predictive model of the world (Dayan et al., 1995; Rao and Ballard, 1999; Friston and Kiebel, 2009; Larochelle and Hinton, 2010; Spratling, 2017; Whittington and Bogacz, 2017; Press et al., 2020). This hypothesis postulates that top-down predictions (i.e., signals from associative regions to sensory regions) are compared to bottom-up signals (i.e., signals from sensory regions to associative regions). Stimuli that violate patterns of past sensory experiences should then induce differences between these two signals.

In line with this postulate, previous experimental work in multiple species and brain regions shows distinct responses to stimuli that either match or violate patterns from past experience (Kumaran and Maguire, 2006; Garrido et al., 2009; Keller et al., 2012; Fiser et al., 2016; Zmarz and Keller, 2016; Orlova et al., 2020; Homann et al., 2022). However, there are still significant unknowns. First, do such responses differ in the distal apical dendrites, where many top-down signals arrive, and the somata, where bottom-up information is likely integrated (Budd, 1998; Jiang et al., 2013; Larkum, 2013; Marques et al., 2018)? There are a few studies that have shown that top-down projections carry distinct, and potentially predictive, information to sensory areas (Fiser et al., 2016; Jordan and Keller, 2020; Orlova et al., 2020), but none have explicitly contrasted the distal apical dendrites and somatic compartments. Second, how do such responses evolve as an animal gains exposure to stimuli that initially violate patterns of past experience, but which become a part of their past experience over increased exposure?

**Figure 1.**
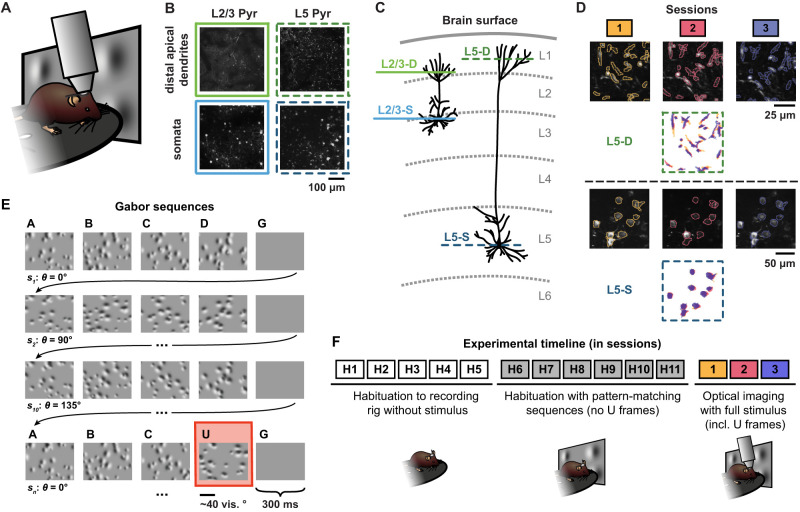
Illustration of experimental methods. ***A***, Experimental setup schematic. Awake, behaving mice were head-fixed under a two-photon microscope objective while passively viewing the stimuli. The mice were able to run freely on a rotating disc. ***B***, Example max-projection images from two-photon recordings for each of the four imaging planes: layer 2/3 distal apical dendrites (L2/3-D), layer 5 distal apical dendrites (L5-D), layer 2/3 somata (L2/3-S), and layer 5 somata (L5-S) (two to three mice per plane, *n* = 11 mice in total; see Materials and Methods). ***C***, Schematic illustration displaying the four imaging planes from *B* within the cortical column. The coloring and style schemes of the horizontal lines depicting the imaging planes here are used throughout all of the figures. ***D***, Tracked region of interest (ROI) examples for both L5-D (*top*) and L5-S (*bottom*). Max-projection images for each imaging session (1, 2, or 3), each performed on a different day, are overlaid with contours of the matched segmented ROI masks. Below the images, the matched ROI masks for all three sessions are superimposed. ***E***, Example Gabor sequences. Each image lasted 300 ms. The mean orientation *θ* of the Gabor patches in each sequence *s*_*i*_ was randomly chosen from $\{ 0^\circ \; 45^\circ \; 90^\circ \; 135^\circ \}$. A pattern-violating image, *U*, with a mean orientation rotated by $90^\circ$ with respect to the other images in the sequence, is highlighted in red. See Materials and Methods and Gillon et al. (2023a) for details. ***F***, Experimental timeline, showing both habituation and imaging sessions. Note that each session occurred on a different day. Optical imaging of neuronal activity was not performed during *H1–H11*.

To address these questions, we performed chronic two-photon calcium imaging at the cell bodies and the distal apical dendrites of layer 2/3 and layer 5 pyramidal neurons in the primary visual cortex of awake, behaving mice over multiple days (Fig. 1*A*–*C*). During the recordings, the animals were exposed to randomly oriented visual stimuli that either matched or violated patterns to which the animals had been habituated. This approach allowed us to track the responses of individual cell bodies and individual distal apical dendritic branches over multiple days (Fig. 1*D*), during which the animals were provided with additional exposure to the pattern-violating stimuli (Fig. 1*E*). We observed interesting differences between the distal apical dendrites and somata. Whereas somatic compartments showed a decrease over days in their differential sensitivity to visual stimuli that either matched or violated the habituated patterns, distal apical dendrites showed an increase in differential sensitivity. This suggests that there may be important differences in the functional roles of the somatic and distal apical dendritic compartments with respect to hierarchical predictive computation or learning stimulus statistics.

## Materials and Methods

The dataset used in this paper was collected as part of the Allen Institute for Brain Science’s OpenScope initiative. We have publicly released the full dataset (Gillon et al., 2023b), and have previously published a dataset descriptor paper without any scientific analysis (Gillon et al., 2023a). In this study, we analyze those data to address questions about neural responses to pattern-violating stimuli. Here, we provide a description of the methods, but additional details can be found within the dataset descriptor paper.

### Imaging and data pre-processing

All animal procedures were approved by the Institutional Animal Care and Use Committee (IACUC) at the Allen Institute for Brain Science, under protocol 1801. To monitor the integration of top-down and bottom-up signals by supra- and sub-granular pyramidal neurons over multiple days, we performed two-photon calcium imaging in awake Cux2-CreERT2 mice or Rbp4-Cre_KL100 mice that expressed GCaMP6f in layer 2/3 or layer 5 pyramidal neurons, respectively (Fig. 1*A*). We performed this imaging either at layer 1 of cortex (50–75 μm depth for layer 2/3 and 20 μm depth for layer 5), thereby observing the distal apical dendrites, or at the layer in which the cell bodies were located (175 μm depth for layer 2/3 and 375 μm depth for layer 5) (Fig. 1*B*–*C*). This gave us four different imaging conditions: layer 2/3 distal apical dendrites (L2/3-D), layer 2/3 somata (L2/3-S), layer 5 distal apical dendrites (L5-D), and layer 5 somata (L5-S). As noted in Gillon et al. (2023a), the L5-S depths are shallower than might otherwise be expected for mouse cortex, as the cranial window pushed gently against the surface of the brain to reduce Z-drift during imaging sessions. GCaMP6f fluorescence tracks calcium influx into cells, but it should be noted that the cause of calcium influx in the somatic and distal apical dendritic compartments may be different, with somatic signals largely reflecting closely-spaced groups of action potentials (Huang et al., 2021) and dendritic signals reflecting a combination of back-propagating action potentials and non-linear dendritic events like NMDA spikes (Murayama et al., 2009). Thus, in both cases we were tracking a proxy for neural activity, but it is important to be aware that the underlying physiological causes of the signal may differ between the two compartments.

Imaging was performed in primary visual cortex (VisP). During the experiments, the animal’s head was fixed in place under the microscope objective, ensuring the stability of our recordings. We extracted regions of interest (ROIs) in each imaging plane (Inan et al., 2017; de Vries et al., 2020; Inan et al., 2021), corresponding to individual distal apical dendrite segments or to individual cell bodies, depending on the imaging plane. Each animal went through three imaging sessions, each performed on a different day, and we used a matching algorithm to identify the same ROIs across sessions (Fig. 1*D*).

Thanks to a conservative quality-control pipeline (de Vries et al., 2020), signal-to-noise ratio (SNR), Δ*F*/*F* magnitudes, and number of ROIs were stable over all three sessions in both layer 2/3 and layer 5 cell bodies and dendrites (Gillon et al., 2023a). Importantly, the ROI extraction algorithm for the dendritic recordings enabled the identification of spatially discontinuous ROIs (Inan et al., 2017, 2021), reducing the risk that single dendritic compartments were split into multiple ROIs. This is supported by the observation that in both the somatic and the dendritic compartments, very few pairs of ROIs showed very high correlations in their responses (see Gillon et al., 2023a). Moreover, while differences in background fluorescence levels were observable between imaging planes (Fig. 1*B*), these did not confound our analyses for two reasons. First, we only compared Δ*F*/*F* levels over days within each imaging plane, not between imaging planes. Second, our analysis pipeline estimated Δ*F*/*F* using a rolling baseline, so that changes in overall fluorescence would not impact our analyses (de Vries et al., 2020).

During these imaging sessions, we tracked the mouse’s movements on a running disc, as well as its pupil diameter with an infrared camera. We obtained calcium imaging data for 11 mice that passed quality control (L2/3-D: *n* = 2, L2/3-S: *n* = 3, L5-D: *n* = 3, L5-S: *n* = 3). The dataset was well-split between female (*n* = 5) and male (*n* = 6) mice, with at least one animal of each sex included in each imaging plane (Gillon et al., 2023a). We tracked ROIs across days using a custom-modified version of the ROI-matching package developed by the Allen Institute for Brain Science (de Vries et al., 2020). The full dataset is freely available online in the DANDI Archive (Gillon et al., 2023b).

### Stimuli

We used two different stimuli for this study: one involving sequences, and one involving visual flow. Our main focus in this paper was a sequential visual stimulus inspired by previous work (Homann et al., 2017, 2022). This stimulus had a predictable global pattern, but stochastic local properties. Thanks to the predictable global pattern we could randomly insert “pattern-violating” events, i.e., stimulus events that violated the predictable global pattern. Mice were exposed to these stimuli over multiple sessions, each occurring on different days, enabling us to observe changes in their neurons’ responses to pattern-matching and pattern-violating sensory events.

#### Gabor sequence stimulus

To build a predictable global pattern with some local stochasticity, we used images composed of randomly placed Gabor patches, assembled into five-image sequences (*A-B-C-D-G*). Other than *G*, which was uniformly gray, each image was defined by the locations of its Gabor patches: e.g., the locations of the Gabor patches were the same for all *A* images for a given session, but differed between *A* and *B* images. These Gabor patch locations were redrawn for each session, and sampled uniformly over the visual field. As a result, the locations were different in each session. Additionally, within each repeat of the sequence (*A-B-C-D-G*), the orientation of each of the Gabor patches was drawn randomly from the same distribution centered around the same mean orientation, but the mean orientation varied from sequence to sequence (Fig. 1*E*). This meant that the luminance patterns at each spatial location were different for each repeat of the *A-B-C-D-G* sequence. However, because all sequences shared a global pattern wherein orientations were drawn from the same distribution across images, a clear pattern is present in the orientations of the Gabors from one image to the next within each sequence. Importantly, given these stimulus design features, the same set of images was never repeated. This reduced the risk of accommodation effects. Nonetheless, the sequences had a predictable global pattern that an observer could identify.

To ensure sufficient exposure to the patterns, before the first calcium imaging session, and after habituation to the rig, the mice were habituated to *A-B-C-D-G* sequences over six sessions, each on a different day, without any violations of the pattern (Fig. 1*F*). After habituation, and during calcium imaging, the stimuli were broken up into approximately 30 blocks of randomly determined durations, each composed of repeated *A-B-C-D-G* sequences, as before. However, instead of comprising only pattern-matching sequences, each block ended with pattern-violation *A-B-C-U-G* sequences. In these sequences, the fourth image, *D*, was replaced with an “un-matching” *U* image, which had different Gabor locations and orientations. Specifically, the newly introduced *U* images had unique random locations and the orientations of the Gabor patches were resampled and shifted by 90$^\circ$ on average with respect to the preceding *A-B-C* images. As such, the *U* images strongly violated the pattern with regard to both Gabor patch locations and orientations. These pattern-violating sequences comprised approximately $7\%$ of the sequences presented to the mice during the imaging sessions (Gillon et al., 2023a).

#### Visual flow stimulus

The visual flow stimulus consisted of 105 white squares moving uniformly across the screen at a velocity of 50 visual degrees per second, with each square being 8 × 8 visual degrees in size. The stimulus was split into two consecutive periods ordered randomly, and each defined by the main direction in which the squares were moving (rightward or leftward, i.e., in the nasal-to-temporal direction or vice versa, respectively). Flow violations were created by reversing the direction of flow of a randomly selected 25% of the squares for 2–4 s at a time, following which they resumed their motion in the main direction of flow (Fig. 6*A*). Similar to the Gabor sequence stimulus, the mice were habituated to the visual flow stimulus without any violations (i.e., with no flow reversals) for six days prior to the recording sessions. During the optical recording sessions, the flow violations were introduced, and they accounted for approximately 5% of the total duration of the visual flow stimulus (Gillon et al., 2023a).

**Table 1.** Summary of statistical tests and results. See Extended Table 1-1 for a spreadsheet version of this table.

Fig.	Panel	Comparison				Bonferroni correction	Corrected *p*-value	Signif.
2	B	L2/3-D	sess. 1 to null		10^5^	24	1.000	n.s.
			sess. 2 to null		permutations	comparisons	1.000	n.s.
			sess. 3 to null				<0.001	*p* < 0.001
			sess. 1 vs 2				<0.001	*p* < 0.001
			sess. 1 vs 3				<0.001	*p* < 0.001
			sess. 2 vs 3				<0.001	*p* < 0.001
						
		L2/3-S	sess. 1 to null				1.000	n.s.
			sess. 2 to null				0.258	n.s.
			sess. 3 to null				1.000	n.s.
			sess. 1 vs 2				1.000	n.s.
			sess. 1 vs 3				1.000	n.s.
			sess. 2 vs 3				0.182	n.s.
						
		L5-D	sess. 1 to null				1.000	n.s.
			sess. 2 to null				<0.001	*p* < 0.001
			sess. 3 to null				<0.001	*p* < 0.001
			sess. 1 vs 2				<0.001	*p* < 0.001
			sess. 1 vs 3				<0.001	*p* < 0.001
			sess. 2 vs 3				0.020	*p* < 0.05
						
		L5-S	sess. 1 to null				<0.001	*p* < 0.001
			sess. 2 to null				1.000	n.s.
			sess. 3 to null				1.000	n.s.
			sess. 1 vs 2				0.755	n.s.
			sess. 1 vs 3				0.011	*p* < 0.05
			sess. 2 vs 3				1.000	n.s.
	
	C	L2/3-D	sess. 1 vs 2	reg.	10^5^	24	1.000	n.s.
				$\text {patt. viol.}$	permutations	comparisons	<0.001	*p* < 0.001
						
			sess. 1 vs 3	reg.			1.000	n.s.
				$\text {patt. viol.}$			<0.001	*p* < 0.001
						
			sess. 2 vs 3	reg.			1.000	n.s.
				$\text {patt. viol.}$			1.000	n.s.
					
		L2/3-S	sess. 1 vs 2	reg.			1.000	n.s.
				$\text {patt. viol.}$			1.000	n.s.
						
			sess. 1 vs 3	reg.			1.000	n.s.
				$\text {patt. viol.}$			<0.001	*p* < 0.001
						
			sess. 2 vs 3	reg.			0.018	*p* < 0.05
				$\text {patt. viol.}$			<0.001	*p* < 0.001
					
		L5-D	sess. 1 vs 2	reg.			1.000	n.s.
				$\text {patt. viol.}$			<0.001	*p* < 0.001
						
			sess. 1 vs 3	reg.			<0.001	*p* < 0.001
				$\text {patt. viol.}$			<0.001	*p* < 0.001
						
			sess. 2 vs 3	reg.			<0.001	*p* < 0.001
				$\text {patt. viol.}$			1.000	n.s.
		L5-S	sess. 1 vs 2	reg.			1.000	n.s.
				$\text {patt. viol.}$			0.814	n.s.
						
			sess. 1 vs 3	reg.			0.118	n.s.
				$\text {patt. viol.}$			1.000	n.s.
						
			sess. 2 vs 3	reg.			1.000	n.s.
				$\text {patt. viol.}$			1.000	n.s.
3	B	L2/3-D	sess. 1		10^5^	12	<0.001	*p* < 0.001
			sess. 2		permutations	comparisons	1.000	n.s.
			sess. 3				<0.001	*p* < 0.001
						
		L2/3-S	sess. 1				1.000	n.s.
			sess. 2				1.000	n.s.
			sess. 3				0.660	n.s.
						
		L5-D	sess. 1				<0.001	*p* < 0.001
(3)	(B)	(L5-D)	sess. 2		(10^5^	(12	<0.001	*p* < 0.001
			sess. 3		permutations)	comparisons)	1.000	n.s.
						
		L5-S	sess. 1				1.000	n.s.
			sess. 2				1.000	n.s.
			sess. 3				0.297	n.s.
4	D	L2/3-D to null			binomial	8	<0.001	*p* < 0.001
	*left*	L2/3-S to null			null CI	comparisons	<0.001	*p* < 0.001
		L5-D to null					<0.001	*p* < 0.001
		L5-S to null					0.004	*p* < 0.01
						
	D	L2/3-D to null					<0.001	*p* < 0.001
	*right*	L2/3-S to null					<0.001	*p* < 0.001
		L5-D to null					<0.001	*p* < 0.001
		L5-S to null					<0.001	*p* < 0.001
						
	E	L2/3-D to null			binomial	8	<0.001	*p* < 0.001
	*left*	L2/3-S to null			null CI	comparisons	<0.001	*p* < 0.001
		L5-D to null					<0.001	*p* < 0.001
		L5-S to null					<0.001	*p* < 0.001
						
	E	L2/3-D to null					<0.001	*p* < 0.001
	*right*	L2/3-S to null					<0.001	*p* < 0.001
		L5-D to null					<0.001	*p* < 0.001
		L5-S to null					<0.001	*p* < 0.001
	
	G	L2/3-D	sess. 1 vs 2		10^5^	12	1.000	n.s.
			sess. 1 vs 3		permutations	comparisons	<0.001	*p* < 0.001
			sess. 2 vs 3				<0.001	*p* < 0.001
						
		L2/3-S	sess. 1 vs 2				0.374	n.s.
			sess. 1 vs 3				<0.001	*p* < 0.001
			sess. 2 vs 3				<0.001	*p* < 0.001
						
		L5-D	sess. 1 vs 2				<0.001	*p* < 0.001
			sess. 1 vs 3				<0.001	*p* < 0.001
			sess. 2 vs 3				1.000	n.s.
						
		L5-S	sess. 1 vs 2				<0.001	*p* < 0.001
			sess. 1 vs 3				0.004	*p* < 0.01
			sess. 2 vs 3				0.683	n.s.
	
	H	L2/3-D	sess. 1 vs 2		10^5^	12	1.000	n.s.
			sess. 1 vs 3		permutations	comparisons	1.000	n.s.
			sess. 2 vs 3				0.647	n.s.
						
		L2/3-S	sess. 1 vs 2				1.000	n.s.
			sess. 1 vs 3				<0.001	*p* < 0.001
			sess. 2 vs 3				<0.001	*p* < 0.001
						
		L5-D	sess. 1 vs 2				0.077	n.s.
			sess. 1 vs 3				0.008	*p* < 0.01
			sess. 2 vs 3				1.000	n.s.
						
		L5-S	sess. 1 vs 2				<0.001	*p* < 0.001
			sess. 1 vs 3				0.002	*p* < 0.01
			sess. 2 vs 3				0.944	n.s.
5	A	L2/3-D	∼6%		binomial	22	<0.001	*p* < 0.001
	*left*		∼24%		null CI	comparisons	<0.001	*p* < 0.001
						
		L2/3-S	∼5%				0.503	n.s.
			∼6%				0.463	n.s.
			∼45%				<0.001	*p* < 0.001
						
		L5-D	∼1%				<0.001	*p* < 0.001
			∼6%				0.095	n.s.
			∼24%				<0.001	*p* < 0.001
						
		L5-S	∼6%				0.542	n.s.
			∼7%				0.330	n.s.
			∼13%				0.250	n.s.
					
(5)	A	L2/3-D	∼2%		(binomial	(22	1.000	n.s.
	*right*		∼9%		null CI)	comparisons)	<0.001	*p* < 0.001
						
		L2/3-S	∼3%				1.000	n.s.
			∼9%				<0.001	*p* < 0.001
			∼51%				<0.001	*p* < 0.001
						
		L5-D	∼1%				0.202	n.s.
			∼6%				0.032	*p* < 0.05
			∼10%				<0.001	*p* < 0.001
						
		L5-S	∼7%				1.000	n.s.
			∼32%				<0.001	*p* < 0.001
			∼34%				<0.001	*p* < 0.001
	
	B	L2/3-D	sess. 1 vs 2	reg.	10^5^	24	1.000	n.s.
				$\text {patt. viol.}$	permutations	comparisons	0.003	*p* < 0.01
						
			sess. 1 vs 3	reg.			0.215	n.s.
				$\text {patt. viol.}$			<0.001	*p* < 0.001
						
			sess. 2 vs 3	reg.			1.000	n.s.
				$\text {patt. viol.}$			1.000	n.s.
					
		L2/3-S	sess. 1 vs 2	reg.			1.000	n.s.
				$\text {patt. viol.}$			1.000	n.s.
						
			sess. 1 vs 3	reg.			0.302	n.s.
				$\text {patt. viol.}$			<0.001	*p* < 0.001
						
			sess. 2 vs 3	reg.			<0.001	*p* < 0.001
				$\text {patt. viol.}$			<0.001	*p* < 0.001
					
		L5-D	sess. 1 vs 2	reg.			1.000	n.s.
				$\text {patt. viol.}$			<0.001	*p* < 0.001
						
			sess. 1 vs 3	reg.			0.050	n.s.
				$\text {patt. viol.}$			<0.001	*p* < 0.001
						
			sess. 2 vs 3	reg.			0.006	*p* < 0.01
				$\text {patt. viol.}$			1.000	n.s.
					
		L5-S	sess. 1 vs 2	reg.			1.000	n.s.
				$\text {patt. viol.}$			0.136	n.s.
						
			sess. 1 vs 3	reg.			<0.001	*p* < 0.001
				$\text {patt. viol.}$			<0.001	*p* < 0.001
						
			sess. 2 vs 3	reg.			1.000	n.s.
				$\text {patt. viol.}$			1.000	n.s.
6	C	L2/3-D	sess. 1 to null		10^5^	24	<0.001	*p* < 0.001
			sess. 2 to null		permutations	comparisons	<0.001	*p* < 0.001
			sess. 3 to null				<0.001	*p* < 0.001
			sess. 1 vs 2				1.000	n.s.
			sess. 1 vs 3				<0.001	*p* < 0.001
			sess. 2 vs 3				<0.001	*p* < 0.001
						
		L2/3-S	sess. 1 to null				0.002	*p* < 0.01
			sess. 2 to null				<0.001	*p* < 0.001
			sess. 3 to null				<0.001	*p* < 0.001
			sess. 1 vs 2				1.000	n.s.
			sess. 1 vs 3				0.132	n.s.
			sess. 2 vs 3				1.000	n.s.
						
		L5-D	sess. 1 to null				1.000	n.s.
			sess. 2 to null				1.000	n.s.
			sess. 3 to null				1.000	n.s.
			sess. 1 vs 2				<0.001	*p* < 0.001
			sess. 1 vs 3				<0.001	*p* < 0.001
			sess. 2 vs 3				<0.001	*p* < 0.001
						
		L5-S	sess. 1 to null				1.000	n.s.
			sess. 2 to null				1.000	n.s.
(6)	(C)	(L5-S)	sess. 3 to null		(10^5^	(24	1.000	n.s.
			sess. 1 vs 2		permutations)	comparisons)	1.000	n.s.
			sess. 1 vs 3				1.000	n.s.
			sess. 2 vs 3				1.000	n.s.
7	A	L2/3-D	sess. 1 vs 2	$\text {unif. flow}$	10^5^	24	0.152	n.s.
				$\text {count. flow}$	permutations	comparisons	1.000	n.s.
						
			sess. 1 vs 3	$\text {unif. flow}$			0.095	n.s.
				$\text {count. flow}$			0.185	n.s.
						
			sess. 2 vs 3	$\text {unif. flow}$			1.000	n.s.
				$\text {count. flow}$			0.003	*p* < 0.01
					
		L2/3-S	sess. 1 vs 2	$\text {unif. flow}$			1.000	n.s.
				$\text {count. flow}$			1.000	n.s.
						
			sess. 1 vs 3	$\text {unif. flow}$			1.000	n.s.
				$\text {count. flow}$			1.000	n.s.
						
			sess. 2 vs 3	$\text {unif. flow}$			1.000	n.s.
				$\text {count. flow}$			1.000	n.s.
					
		L5-D	sess. 1 vs 2	$\text {unif. flow}$			<0.001	*p* < 0.001
				$\text {count. flow}$			<0.001	*p* < 0.001
						
			sess. 1 vs 3	$\text {unif. flow}$			<0.001	*p* < 0.001
				$\text {count. flow}$			0.057	n.s.
						
			sess. 2 vs 3	$\text {unif. flow}$			1.000	n.s.
				$\text {count. flow}$			0.344	n.s.
					
		L5-S	sess. 1 vs 2	$\text {unif. flow}$			0.072	n.s.
				$\text {count. flow}$			0.067	n.s.
						
			sess. 1 vs 3	$\text {unif. flow}$			1.000	n.s.
				$\text {count. flow}$			1.000	n.s.
						
			sess. 2 vs 3	$\text {unif. flow}$			1.000	n.s.
				$\text {count. flow}$			0.621	n.s.
	
	B	L2/3-D	gabors vs vis. flow		10^5^	5	<0.001	*p* < 0.001
		L2/3-S	gabors vs vis. flow		permutations	comparisons	<0.001	*p* < 0.001
		L5-D	gabors vs vis. flow				<0.001	*p* < 0.001
		L5-S	gabors vs vis. flow				1.000	n.s.
		all	gabors vs vis. flow				<0.001	*p* < 0.001
	
	D	L2/3-D	sess. 1 vs 2		10^5^	12	1.000	n.s.
			sess. 1 vs 3		permutations	comparisons	0.034	*p* < 0.05
			sess. 2 vs 3				0.147	n.s.
						
		L2/3-S	sess. 1 vs 2				1.000	n.s.
			sess. 1 vs 3				<0.001	*p* < 0.001
			sess. 2 vs 3				0.026	*p* < 0.05
						
		L5-D	sess. 1 vs 2				1.000	n.s.
			sess. 1 vs 3				1.000	n.s.
			sess. 2 vs 3				0.588	n.s.
						
		L5-S	sess. 1 vs 2				1.000	n.s.
			sess. 1 vs 3				1.000	n.s.
			sess. 2 vs 3				1.000	n.s.
	
	E	L2/3-D	gabors vs vis. flow		10^5^	5	0.650	n.s.
		L2/3-S	gabors vs vis. flow		permutations	comparisons	1.000	n.s.
		L5-D	gabors vs vis. flow				0.005	*p* < 0.01
		L5-S	gabors vs vis. flow				0.243	n.s.
		all	gabors vs vis. flow				<0.001	*p* < 0.001
8	B	L2/3-D to null			10^4^	4	1.000	n.s.
	*left*	L2/3-S to null			permutations	comparisons	1.000	n.s.
		L5-D to null					1.000	n.s.
		L5-S to null					1.000	n.s.
	
	B	L2/3-D to null			10^4^	4	1.000	n.s.
	*right*	L2/3-S to null			permutations	comparisons	0.782	n.s.
		L5-D to null					1.000	n.s.
		L5-S to null					1.000	n.s.
9	C	L2/3-D	sess. 1 vs 2 to null		10^5^	8	<0.001	*p* < 0.001
			sess. 2 vs 3 to null		permutations	comparisons	1.000	n.s.
						
		L2/3-S	sess. 1 vs 2 to null		(lower tail)		0.843	n.s.
			sess. 2 vs 3 to null				1.000	n.s.
						
		L5-D	sess. 1 vs 2 to null				<0.001	*p* < 0.001
			sess. 2 vs 3 to null				1.000	n.s.
						
		L5-S	sess. 1 vs 2 to null				1.000	n.s.
			sess. 2 vs 3 to null				1.000	n.s.
	
	F	L2/3-D	sess. 1 vs 2 to null		10^5^	8	1.000	n.s.
			sess. 2 vs 3 to null		permutations	comparisons	1.000	n.s.
						
		L2/3-S	sess. 1 vs 2 to null		(lower tail)		1.000	n.s.
			sess. 2 vs 3 to null				1.000	n.s.
						
		L5-D	sess. 1 vs 2 to null				1.000	n.s.
			sess. 2 vs 3 to null				1.000	n.s.
						
		L5-S	sess. 1 vs 2 to null				1.000	n.s.
			sess. 2 vs 3 to null				1.000	n.s.
10	A	L2/3-D	sess. 1 to null		10^5^	60	1.000	n.s.
			sess. 2 to null		permutations	comparisons	1.000	n.s.
			sess. 3 to null		(upper tail)		1.000	n.s.
						
		L2/3-S	sess. 1 to null				<0.001	*p* < 0.001
			sess. 2 to null				0.005	*p* < 0.01
			sess. 3 to null				0.017	*p* < 0.05
						
		L5-D	sess. 1 to null				0.337	n.s.
			sess. 2 to null				1.000	n.s.
			sess. 3 to null				1.000	n.s.
						
		L5-S	sess. 1 to null				0.706	n.s.
			sess. 2 to null				1.000	n.s.
			sess. 3 to null				1.000	n.s.
					
	B	L2/3-D	sess. 1 to null				1.000	n.s.
			sess. 2 to null				0.232	n.s.
			sess. 3 to null				0.127	n.s.
						
		L2/3-S	sess. 1 to null				<0.001	*p* < 0.001
			sess. 2 to null				<0.001	*p* < 0.001
			sess. 3 to null				<0.001	*p* < 0.001
						
		L5-D	sess. 1 to null				0.008	*p* < 0.01
			sess. 2 to null				0.020	*p* < 0.05
			sess. 3 to null				0.063	n.s.
						
		L5-S	sess. 1 to null				0.032	*p* < 0.05
			sess. 2 to null				0.509	n.s.
			sess. 3 to null				0.085	n.s.
					
	C	L2/3-D	sess. 1 to null				0.471	n.s.
			sess. 2 to null				0.002	*p* < 0.01
			sess. 3 to null				0.116	n.s.
						
		L2/3-S	sess. 1 to null				<0.001	*p* < 0.001
			sess. 2 to null				<0.001	*p* < 0.001
			sess. 3 to null				<0.001	*p* < 0.001
						
(10)	(C)	L5-D	sess. 1 to null		(10^5^	(60	0.029	*p* < 0.05
			sess. 2 to null		permutations	comparisons)	<0.001	*p* < 0.001
			sess. 3 to null		(upper tail))		0.004	*p* < 0.01
		L5-S	sess. 1 to null				0.092	n.s.
			sess. 2 to null				0.159	n.s.
			sess. 3 to null				<0.001	*p* < 0.001
					
	D	L2/3-D	sess. 1 to null				<0.001	*p* < 0.001
			sess. 2 to null				0.002	*p* < 0.01
			sess. 3 to null				0.114	n.s.
						
		L2/3-S	sess. 1 to null				<0.001	*p* < 0.001
			sess. 2 to null				<0.001	*p* < 0.001
			sess. 3 to null				<0.001	*p* < 0.001
						
		L5-D	sess. 1 to null				0.001	*p* < 0.01
			sess. 2 to null				0.012	*p* < 0.05
			sess. 3 to null				0.011	*p* < 0.05
						
		L5-S	sess. 1 to null				0.001	*p* < 0.01
			sess. 2 to null				1.000	n.s.
			sess. 3 to null				<0.001	*p* < 0.001
					
	E	L2/3-D	sess. 1 to null				0.439	n.s.
			sess. 2 to null				<0.001	*p* < 0.001
			sess. 3 to null				0.002	*p* < 0.01
						
		L2/3-S	sess. 1 to null				<0.001	*p* < 0.001
			sess. 2 to null				<0.001	*p* < 0.001
			sess. 3 to null				0.004	*p* < 0.01
						
		L5-D	sess. 1 to null				0.037	*p* < 0.05
			sess. 2 to null				<0.001	*p* < 0.001
			sess. 3 to null				<0.001	*p* < 0.001
						
		L5-S	sess. 1 to null				<0.001	*p* < 0.001
			sess. 2 to null				0.473	n.s.
			sess. 3 to null				0.047	*p* < 0.05
11	A	L2/3-D	*D* sequences		10^4^	8	2/45 pts	*p* < 0.05
			*U* sequences		permutations	comparisons	0/45 pts	*p* < 0.05
						
		L2/3-S	*D* sequences		(upper tail)		40/45 pts	*p* < 0.05
			*U* sequences				36/45 pts	*p* < 0.05
						
		L5-D	*D* sequences				0/45 pts	*p* < 0.05
			*U* sequences				4/45 pts	*p* < 0.05
						
		L5-S	*D* sequences				5/45 pts	*p* < 0.05
			*U* sequences				11/45 pts	*p* < 0.05
	
	B	L2/3-D	*D* sequences		10^4^	8	0/45 pts	*p* < 0.05
			*U* sequences		permutations	comparisons	2/45 pts	*p* < 0.05
						
		L2/3-S	*D* sequences		(upper tail)		20/45 pts	*p* < 0.05
			*U* sequences				19/45 pts	*p* < 0.05
						
		L5-D	*D* sequences				3/45 pts	*p* < 0.05
			*U* sequences				6/45 pts	*p* < 0.05
						
		L5-S	*D* sequences				0/45 pts	*p* < 0.05
			*U* sequences				0/45 pts	*p* < 0.05
	
	C	L2/3-D	*D* sequences		10^4^	8	0/45 pts	*p* < 0.05
			*U* sequences		permutations	comparisons	2/45 pts	*p* < 0.05
						
		L2/3-S	*D* sequences		(upper tail)		20/45 pts	*p* < 0.05
			*U* sequences				18/45 pts	*p* < 0.05
						
		L5-D	*D* sequences				0/45 pts	*p* < 0.05
			*U* sequences				13/45 pts	*p* < 0.05
						
		L5-S	*D* sequences				6/45 pts	*p* < 0.05
			*U* sequences				1/45 pts	*p* < 0.05

10.1523/JNEUROSCI.2324-22.2023.t1-1 Table 1-1 Download Table 1-1, XLS file.

### Analysis of locomotion and pupil diameter

Mice were head-fixed and placed on a disc on which they were free to run for both habituation and imaging sessions. Running velocity was converted from disc rotations per frame to cm/s. The velocities obtained were then median-filtered using a kernel size of five frames. Any outliers, which were identified using a single frame velocity change of >±50 cm/s, were omitted from subsequent analysis.

Pupil diameter was tracked using an infrared LED on the ipsilateral eye (relative to the monitor), allowing the recording of infrared video (Allen Institute for Brain Science, 2017). Using DeepLabCut (Mathis et al., 2018), we manually labeled ∼200 examples to train the algorithm to automatically identify a constellation of points around the pupil. We then estimated the pupil diameter (∼0.01 mm per pixel conversion) from these points (see code on GitHub: https://allensdk.readthedocs.io/en/latest/allensdk.internal.brain_observatory.eye_calibration.html). Again, outliers, defined here as single-frame diameter changes of at least 0.05 mm, were omitted (as these appeared to be due to blinking). For more details on the pre-processing of running velocity and pupil diameter data, see Gillon et al. (2023a).

Each datapoint in Figure 8*B* corresponds to the difference in the mean running velocity or pupil diameter for one block between the pattern-violating and preceding pattern-matching Gabor sequences during session 1, with all blocks being pooled across mice. We computed *p*-values by comparing the mean difference over all blocks for each plane to a distribution of mean differences obtained by pairwise shuffling the pattern-violation and pattern-matching labels 10^4^ times and calculating the mean difference over all blocks for each shuffle.

### Fluorescence trace analysis

**Figure 2.**
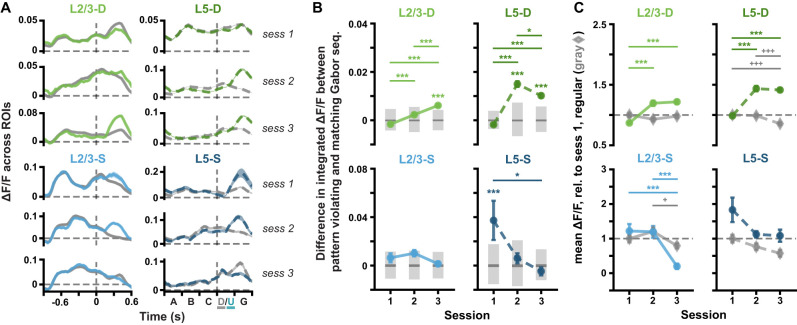
Δ*F*/*F* responses to pattern-violating stimuli evolve over days. ***A***, Mean (± SEM) across ROI mean Δ*F*/*F* responses to pattern-matching (gray, *A-B-C-D-G*) and pattern-violating (green or blue, *A-B-C-U-G*) Gabor sequences. Dashed vertical lines mark onset of *D*/*U* images. ***B***, Mean (± SEM) differences across ROIs in the mean integrated responses to pattern-matching vs pattern-violating Gabor sequences, as defined in *A*. Gray bars show median (dark) and adjusted 95% CIs (light) over differences expected by chance. ***C***, Mean (± SEM) across ROIs of the mean Δ*F*/*F* responses across sequences for regular sequence images (gray diamonds: *A-B-C*) and pattern-violating images (green or blue circles: *U-G*). Responses are calculated relative to session 1 regular responses, marked by dashed horizontal lines. *n* ROIs in sess. 1/2/3: L2/3-D (1,355/648/845), L2/3-S (407/388/397), L5-D: (2,807/1,633/1,664), L5-S (204/183/205). *p < 0.05, **p < 0.01, ***p < 0.001 (two-tailed, corrected). ^+^*p* < 0.05, ^++^*p* < 0.01, ^+++^*p*< 0.001 (two-tailed, corrected), for regular stimulus comparisons (gray) in C. See Table 1 for details of statistical tests and precise *p*-values for all comparisons.

For all results except those presented in Figures 5*A*, *C*, 10, and 11, ROIs were pooled across all mice within an imaging plane for analyses. To enable ROI pooling across mice within imaging planes, each ROI’s Δ*F*/*F* trace was scaled using robust standardization, i.e., by subtracting the median and then dividing by the inter-percentile range spanning the 5^th^ to 95^th^ percentile. The only additional exceptions to this are Figures 2*C* and 7a, where unscaled Δ*F*/*F* traces were used to ascertain how the Δ*F*/*F* signal itself changed across sessions. We note that there was no evidence for any changes over days as a result of bleaching or tissue health as the overall signals did not change in magnitude or SNR over days (Gillon et al., 2023a).

**Figure 3.**
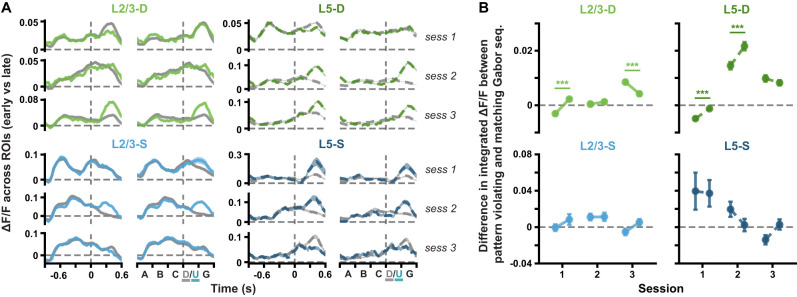
(*Extended analysis supporting Fig. 2.*) Δ*F*/*F* responses to pattern-violating stimuli evolve within sessions in the distal apical dendrites. ***A***, Mean (± SEM) across ROI mean Δ*F*/*F* responses to pattern-matching (gray, *A-B-C-D-G*) and pattern-violating (green or blue, *A-B-C-U-G*) Gabor sequences in the first third (early part, *left*) and last third (late part, *right*) of each session. Dashed vertical lines mark onset of *D*/*U* images. ***B***, Mean (± SEM) differences across ROIs in the mean integrated responses to pattern-matching vs pattern-violating Gabor sequences, as defined in *A*, for the first and last third of each session (early and late parts, respectively). Dashed horizontal lines mark a difference of 0. Statistical significance was evaluated within each session, by comparing the differences computed for the early part vs the late part of a session. The difference in response to *D* vs *U* images changes within sessions in the distal apical dendrites, specifically within sessions 1 and 3 for L2/3, and within sessions 1 and 2 for L5. *p < 0.05, **p < 0.01, ***p < 0.001 (two-tailed, corrected). See Table 1 for details of statistical tests and precise *p*-values for all comparisons.

For Figure 2, *A* and *B*, ROI responses and differences in responses to full pattern-matching (*A-B-C-D-G*) and pattern-violating (*A-B-C-U-G*) sequences were obtained by first taking the mean Δ*F*/*F* for each ROI across Gabor sequences. Mean Δ*F*/*F* ± standard error of the mean (SEM) traces were then computed across ROIs and plotted for each session and imaging plane (Fig. 2*A*). The same analysis was run for Figure 3*A*, but in this case, statistics were computed using only the responses to sequences from the first third of each session (early part, *left*) or the last third (late part, *right*). For Figure 2*B*, the differences in the traces plotted in Figure 2*A* were quantified by integrating the mean Δ*F*/*F* responses over time for each ROI. Mean differences ± SEM between pattern-matching and pattern-violating sequence responses were then calculated across ROIs and plotted for each session and imaging plane. Similarly, for Figure 3*B*, differences were computed separately for responses to sequences in the first third (early part) and the last third (late part) of each session. To further compare ROI responses to the pattern-matching vs pattern-violating stimuli, we also considered the regular, always pattern-matching, component of the Gabor sequences (*A-B-C*), and compared it to the pattern-violating (*U-G*) stimuli. Specifically, for each ROI, a mean Δ*F*/*F* was calculated for each set of Gabor images, and then across sequences (*A-B-C* vs *U-G*, Fig. 2*C*). The mean Δ*F*/*F* values thus obtained for each ROI over a given session were then normalized by dividing by the mean Δ*F*/*F* for regular stimuli (*A-B-C*) across all ROIs from the same mouse in session 1. These normalized means ± SEM over ROIs were then plotted for each session and plane.

**Figure 4.**
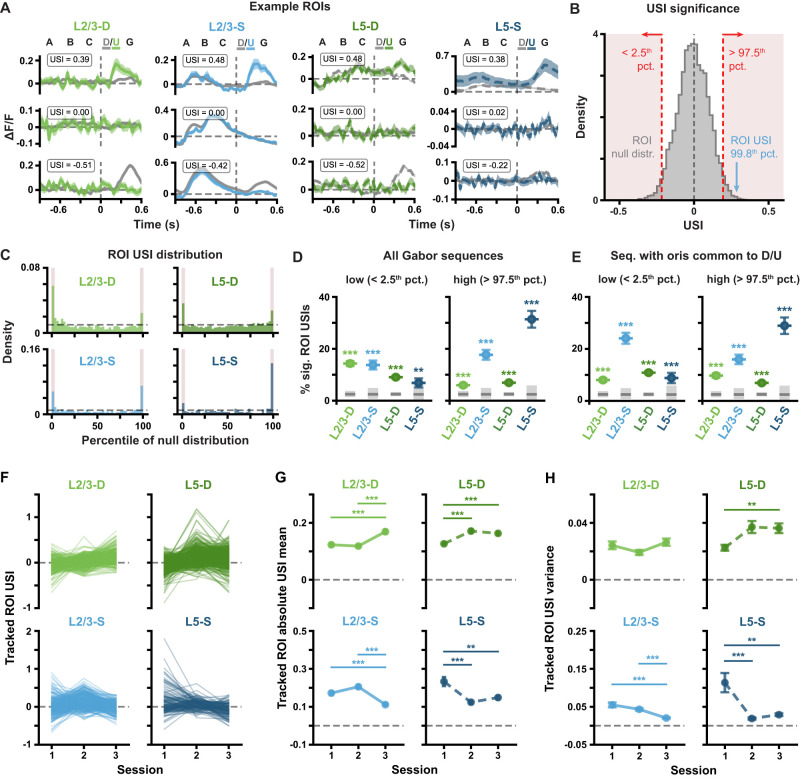
Pattern-violating Gabor sequences result in different Δ*F*/*F* and USI changes in different imaging planes. ***A***, Example session 1 Δ*F*/*F* response traces for individual compartment (*left* to *right*: L2/3-D, L2/3-S, L5-D and L5-S) ROIs with high (*top*), null (*middle*) or low (*bottom*) USIs. Mean ± standard error of the mean (SEM) Δ*F*/*F* across Gabor sequences is plotted. Dashed vertical lines mark onset of *D*/*U* images. ***B***, Example USI null distribution for one ROI from L2/3-S in session 1, generated by shuffling *D-G* and *U-G* labels for the same ROI and recomputing the shuffled USIs 10^4^ times. Significant regions highlighted in red, and true USI value labeled in blue. ***C***, USI percentile distributions for each plane for all session 1 ROIs. Dashed horizontal lines depict null hypotheses (i.e., uniform distribution). Significant regions highlighted in red (*p* < 0.05, shuffle test as shown in *B*). ***D***, Percentage ± bootstrapped standard deviation (SD) of significant USIs for all segmented ROIs in session 1 for each plane. All sequences (any mean orientation) are included in the analysis. ***E***, Same as *D*, but restricted to Gabor sequences with mean orientations shared between *D* and *U* images $\{ 90^\circ \; 135^\circ \}$. ***F***, Gabor sequence stimulus USIs for all tracked ROIs. Each line represents a single ROI’s USIs over all three sessions. ***G***, Mean (± SEM) across the absolute values of the Gabor sequence stimulus USIs for tracked ROIs, as shown in *F*. ***H***, Variance (± bootstrapped SD) across the Gabor sequence stimulus USIs for tracked ROIs, as shown in *F*. *p < 0.05, **p < 0.01, ***p < 0.001 (two-tailed, corrected). See Table 1 for details of statistical tests and precise *p*-values for all comparisons.

“Un-matching event” selectivity indices (USIs) (Fig. 4) were calculated for each ROI separately using Equation 1:

(1) $$\text{USI}\equals {\mu_{{\rm violating}} - \mu_{{\rm matching}}\over \sqrt{\frac{1}{2}\left(\sigma_{{\rm violating}}^2 \plus \sigma_{{\rm matching}}^2\right)}}\;$$ (0.1)

where the means ($\mu _{{\rm matching}}$ and $\mu _{{\rm violating}}$) and variances ($\sigma _{{\rm matching}}^2$ and $\sigma _{{\rm violating}}^2$) were calculated across integrated Δ*F*/*F* responses to the pattern-matching and pattern-violating stimulus events, respectively. For the Gabor sequences, pattern-matching event responses were defined as those spanning *D-G* images, and pattern-violating events were defined as those spanning *U-G* images, with each event therefore spanning 600 ms. Indeed, *G* images were included in these events, as they did not introduce any new stimuli, but did consistently show persisting ROI responses to *D* or *U* images (Figs. 2*A*, 4*A*). For each ROI, in addition to the true USI, a null distribution over USIs was obtained by randomly reassigning the pattern-matching and pattern-violating event labels to each response 10^4^ times. USIs were deemed significantly low if they lay below the 2.5^th^ percentile, and significantly high if they lay above the 97.5^th^ percentile of their null distribution (Fig. 4*B*).

Note that for Figure 4*E*, USIs were calculated using only *D-G* and *U-G* stimuli for which the mean orientations were in $\{ 90^\circ \; 135^\circ \}$, i.e., the orientations shared by *D* and *U* images. For each imaging plane, the percentage of significant ROI USIs was then plotted with bootstrapped standard deviations (SDs). Adjusted 95% CIs over chance levels were estimated using the usual approximation method of the binomial CI, with the sample size corresponding to the number of ROIs in the plane (Fig. 4*D*–*E*).

Absolute fractional differences between sessions in the responses to pattern-violating stimuli (Fig. 7*B*) or in USIs (Fig. 7*E*) were defined as:

(2) $$\left\vert {\mu_j - \mu_i\over \mu_i} \right\vert \;$$ (0.2)

where the subscripts {*i*, *j*} ∈ {1, 2, 3} indicate the session over which the mean *μ* is computed.

**Figure 5.**
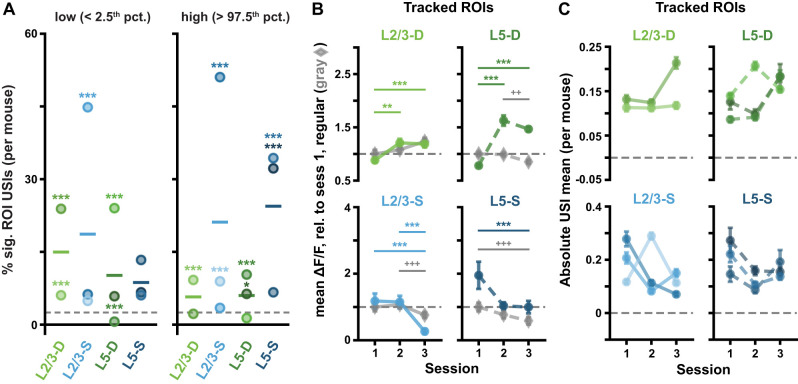
(*Extended analysis supporting Fig. 4.*) ROI responses to pattern-violating Gabor sequences are consistent in tracked ROIs and across mice. ***A***, Percentage of significant USIs in session 1 for each plane, where each dot corresponds to a separate mouse. Statistical significance for each datapoint was evaluated against its own adjusted binomial CI (not shown). Lines show the pooled percentage across mice for each plane (slightly different from the pooled percentage across ROIs plotted in Fig. 4*D*). Dashed horizontal lines mark the theoretical chance level (2.5%). Results are consistent with those pooled across mice, with 10 out of the 11 animals showing a higher percentage of significant ROI USIs than expected by chance in at least one tail (Fig. 4*D*). ***B***, Mean (± SEM) across tracked ROIs of the mean Δ*F*/*F* responses across sequences for regular sequence pattern-matching images (gray diamonds: *A-B-C*) and pattern-violating images (green or blue circles, *U-G*), as in Fig. 2*C*. Responses are calculated relative to session 1 regular sequence responses, marked by dashed horizontal lines. Results are consistent with the full ROI population results (Fig. 2*C*). ***C***, Mean (± SEM) across the absolute values of the Gabor sequence stimulus USIs for tracked ROIs, as in Figure 4*G*, but split by mouse. Results are consistent with those pooled across mice (Fig. 4*G*). *p < 0.05, **p< 0.01, ***p < 0.001 (two-tailed, corrected). ^+^*p* < 0.05, ^++^*p* < 0.01, ^+++^*p* < 0.001 (two-tailed, corrected), for regular stimulus comparisons (gray) in *B*. See Table 1 for details of statistical tests and precise *p*-values for all comparisons.

**Figure 6.**
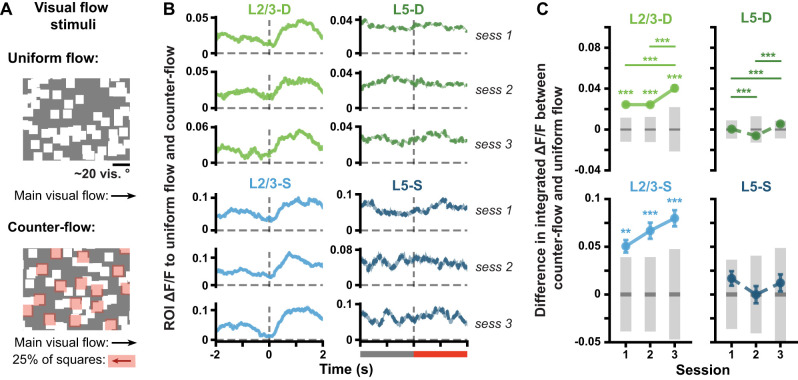
Within each cortical layer, somata and apical dendrites respond similarly to the onset of counter-flow. ***A***, Visual flow stimulus. Squares moved together at the same velocity across the screen during uniform flow (*top*). At random times (counter-flow, *bottom*), 25% of the squares, highlighted here in red for illustrative purposes, reversed direction for 2–4 s (see Materials and Methods). ***B***, Mean (± SEM) across ROI mean Δ*F*/*F* responses to visual flow sequences. Uniform and counter-flow visual flow sequences were defined as for the USI calculation, namely over the 2 s preceding counter-flow onset and following its onset, respectively. Dashed vertical line at time 0 marks the onset of counter-flow, also signaled by the gray bar becoming red (bottom of right column). ***C***, Mean (± SEM) differences across ROIs in the mean integrated responses to uniform flow vs counter-flow, as defined in *B*. Gray bars show median (dark) and adjusted 95% CIs (light) over randomly expected differences. *p < 0.05, **p < 0.01, ***p < 0.001 (two-tailed, corrected). See Table 1 for details of statistical tests and precise *p*-values for all comparisons.

**Figure 7.**
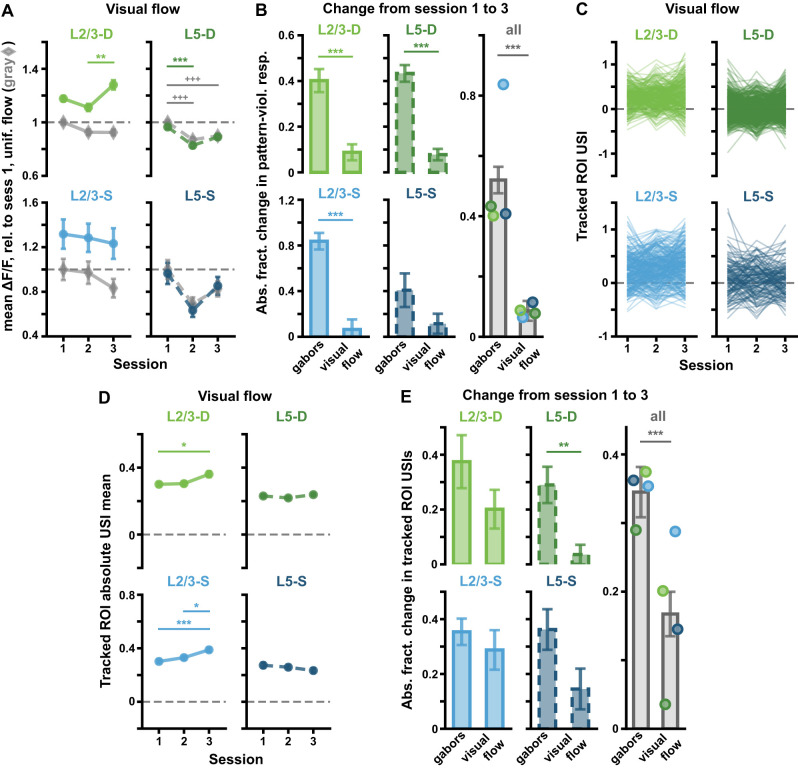
(*Extended analysis supporting Fig. 6.*) Visual counter-flow sequences do not result in the same Δ*F*/*F* changes across sessions as do pattern-violating Gabor sequences. ***A***, Mean (± SEM) across ROIs of the mean Δ*F*/*F* responses across sequences for uniform flow (gray diamonds) and counter-flow (green or blue circles), as defined in Fig. 6*B*. Responses are calculated relative to session 1 uniform flow responses, marked by dashed horizontal lines. Corresponds to Fig. 2*C* for Gabor sequences. ***B***, Absolute fractional change (± bootstrapped SD) in mean responses to pattern-violating stimuli from session 1 to 3 for Gabor sequences vs the counter-flow stimulus for each plane (*left* and *middle* columns), and pooled across all planes (*right* column) (see Eq. 2). In all imaging planes except L5-S, changes in ROI responses to the pattern-violating Gabor stimulus from session 1 to 3 were significantly greater than for the visual counter-flow stimulus. ***C***, Visual flow stimulus USIs for all tracked ROIs. Each line represents a single ROI’s USIs over all three sessions. Corresponds to Fig. 4*F* for Gabor sequences. ***D***, Mean (± SEM) across the absolute values of the visual flow stimulus USIs for tracked ROIs, as shown in *C*. Corresponds to Fig. 4*G* for Gabor sequences. ***E***, Similar to *B*, but here mean (± bootstrapped SD) absolute fractional changes in USIs from session 1 to 3 across tracked ROIs are plotted (see Eq. 2). In L5-D and all compartments combined, changes in USIs for tracked ROIs from session 1 to 3 were significantly greater for the Gabor stimulus than for the visual flow stimulus. *p < 0.05, **p < 0.01, ***p < 0.001 (two-tailed, corrected). ^+^*p* < 0.05, ^++^*p* < 0.01, ^+++^*p* < 0.001 (two-tailed, corrected), for uniform pattern stimulus comparisons (gray) in *A*. See Table 1 for details of statistical tests and precise *p*-values for all comparisons.

Pearson correlation coefficients, and the corresponding regression slopes (Fig. 9) were calculated to compare ROI USIs in each imaging plane between sessions. Bootstrapped SDs over these correlations for each plane were then estimated, and adjusted 95% CIs were computed by permuting the ROI labels, such that tracked ROIs were no longer matched together. Here, one-tailed (lower tail) CIs were calculated to identify correlations that were more negative than expected by chance.

**Figure 8.**
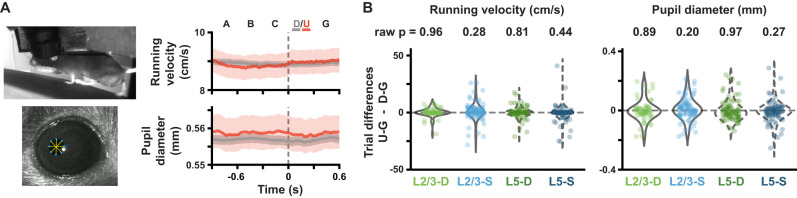
Absence of behavioral responses to pattern-violating stimuli. ***A***, (*Left*) Example frames of a mouse running (*top*), and of a mouse pupil with tracking markers (*bottom*). (*Right*) Running velocity and pupil diameter traces aggregated across mice (mean ± SEM across Gabor sequences) for pattern-matching (gray) and pattern-violating (red) sequences. Note that the smaller SEM is due to the greater number of pattern-matching sequences, compared to pattern-violating ones. Dashed vertical lines mark onset of D/U images. ***B***, Block-by-block running velocity (*left*) and pupil diameter (*right*) differences between pattern-violating (*U-G*) and pattern-matching (*D-G*) images. Raw two-tailed *p*-values (not corrected for multiple comparisons) are printed in the panel, whereas corrected *p*-values are reported in Table 1. *p < 0.05, **p < 0.01, ***p < 0.001. See Table 1 for details of statistical tests and precise *p*-values for all comparisons.

**Figure 9.**
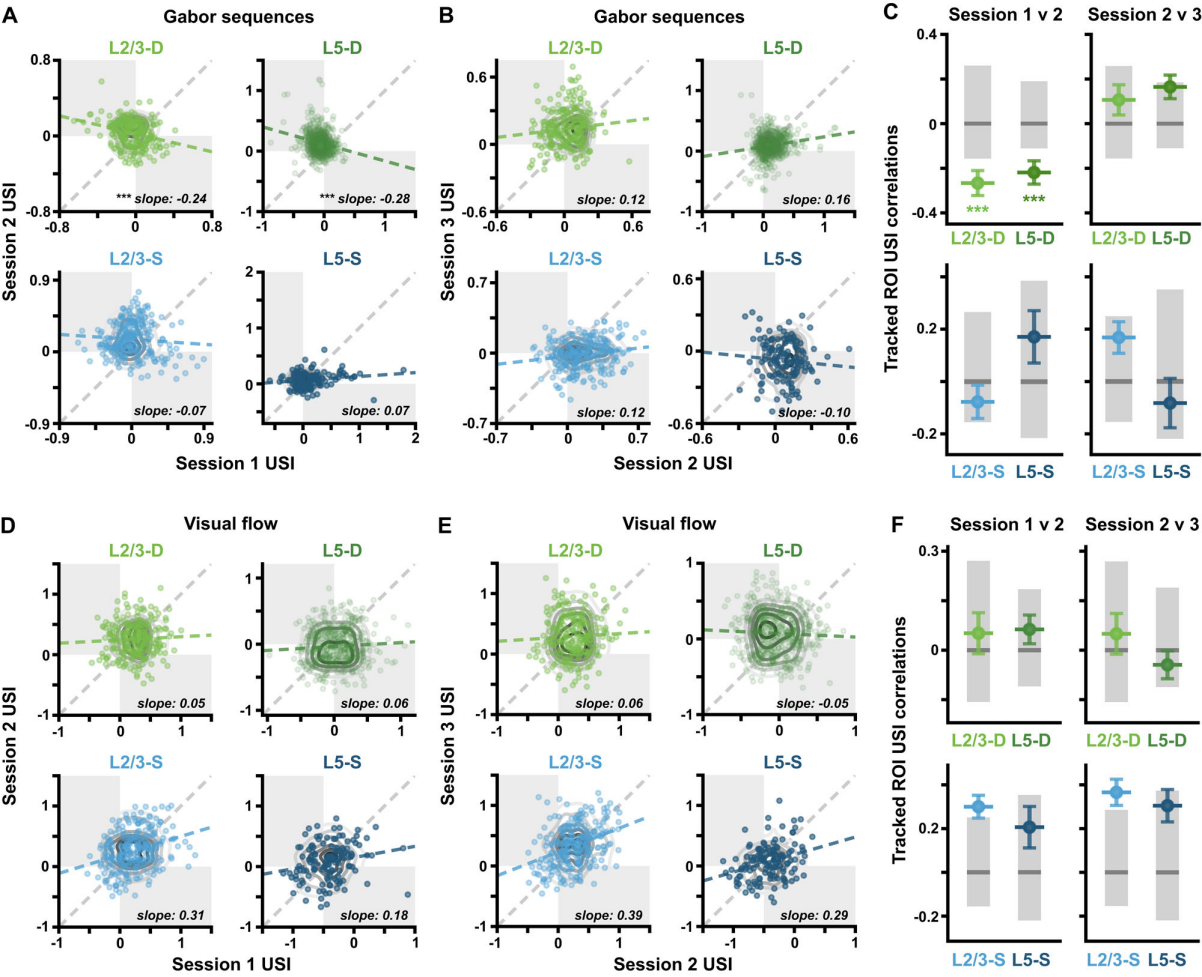
Pattern-violating Gabor sequences result in predictable Δ*F*/*F* changes in distal apical dendritic ROIs. ***A***, Gabor USI scatterplots showing correlations between sessions 1 and 2. Each point reflects a single-tracked ROI’s USIs on two sessions. Gray contour lines show null distributions, computed by shuffling ROI labels. The estimated regression slopes for each plane (blue or green, dashed) are plotted against the identity line (gray, dashed). Opposite quadrants are shaded in gray. ***B***, Same as in *A*, but for Gabor sequence USIs in sessions 2 and 3. ***C***, USI correlations (± bootstrapped SD) from *A* and *B*. Gray bars show median (dark) and adjusted 95% CIs (light), computed by shuffling ROI labels. ***D***, Same as in *A*, but for visual flow USIs in sessions 1 and 2. ***E***, Same as in *A*, but for visual flow USIs in sessions 2 and 3. ***F***, Same as in *C*, but for visual flow USI correlations. *p < 0.05, **p < 0.01, ***p < 0.001 (one-tailed (lower), corrected). See Table 1 for details of statistical tests and precise *p*-values for all comparisons.

**Figure 10.**
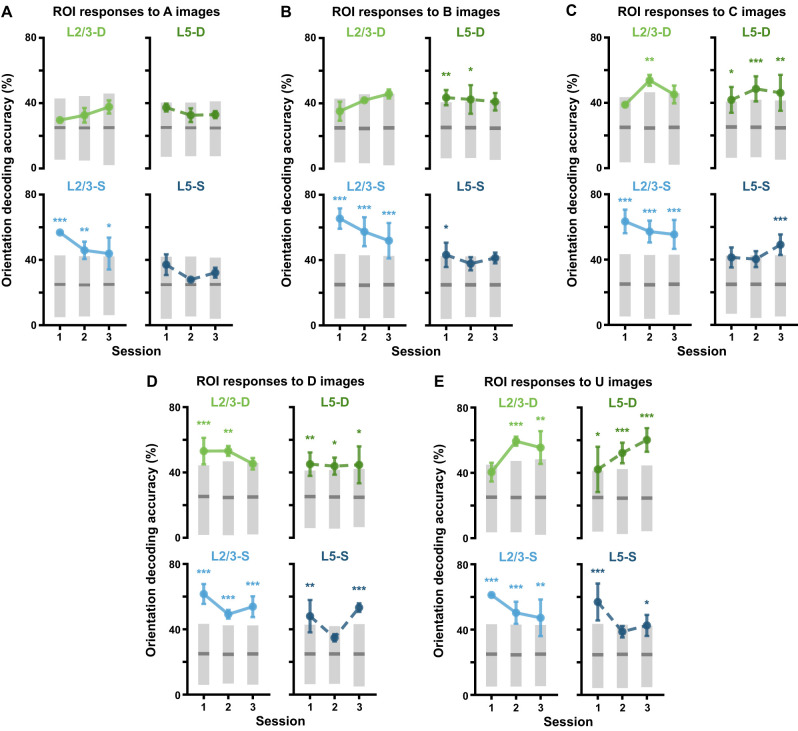
Stimulus properties are decodable across sessions. ***A***, Balanced accuracy (mean ± SEM over mice) for classifiers decoding mean Gabor patch orientations from ROI activity during *A* images viewed during *A-B-C-D-G* sequences (2–3 mice per imaging plane, 300 random cross-validation splits per mouse, per session). Gray bars show median (dark) and adjusted one-tailed 95% CIs (light), computed by shuffling orientation labels. ***B***, Same as *A* but with ROI activity during *B* images viewed during *A-B-C-D-G* sequences. ***C***, Same as *A* but with ROI activity during *C* images viewed during *A-B-C-D-G* sequences. ***D***, Same as *A* but with ROI activity during *D* images viewed during *A-B-C-D-G* sequences. ***E***, Same as *A* but with ROI activity during *U* images viewed during *A-B-C-U-G* sequences. *p < 0.05, **p < 0.01, ***p < 0.001 (one-tailed (upper), corrected).See Table 1 for details of statistical tests and precise *p*-values for all comparisons.

For the orientation decoding analyses (Fig. 10), logistic regressions were trained with an ℓ^2^ penalty on the multinomial task of classifying the mean Gabor patch orientation for *A*, *B*, *C*, or *D* images $\{ 0^\circ \; 45^\circ \; 90^\circ \; 135^\circ \}$ from pattern-matching sequences (*A-B-C-D-G*) or for *U* images $\{ 90^\circ \; 135^\circ \; 180^\circ \; 225^\circ \}$ from pattern-violating sequences (*A-B-C-U-G*). Balanced classifier accuracy was evaluated on the test sets of 300 random cross-validation 75:25 train:test splits of the dataset for each mouse. Importantly, since the *A*, *B*, *C*, and *D* image datasets necessarily comprised many more examples than the *U* image datasets (∼13x), they were first downsampled for each split to match the number of examples in the corresponding *U* image datasets, thus enabling fairer comparisons between the orientation classification results for the different images. Input data consisted of the frame-concatenated Δ*F*/*F* responses for all ROIs together from the onset of each image to 450 ms after. The input window was chosen to extend beyond the 300 ms duration of each image in order to take into account the extended responses to *D* and *U* images, as mentioned above in the USI definition. The traces were standardized as described above, but using statistics drawn from the training data only. Mean balanced accuracy across dataset splits was calculated for each mouse, and the mean (± SEM) balanced accuracy across mice was plotted for each session and plane. To estimate chance accuracy, shuffled classifier performances were evaluated on 10^5^ random cross-validation dataset splits for each mouse. These classifiers were trained as above, but for each split, the training set orientation targets were shuffled randomly. Null distributions over mean performance were obtained by averaging classifier accuracy for each split across mice, from which adjusted one-tailed 95% CIs (upper tail) over accuracy levels expected by chance were calculated for each session and plane.

**Figure 11.**
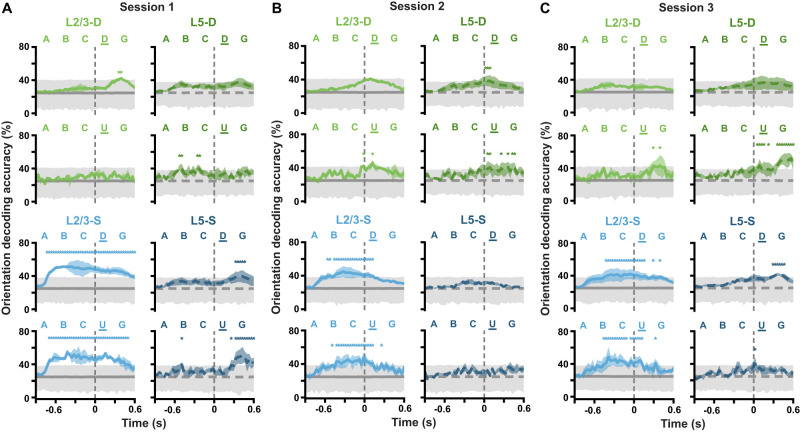
(*Extended analysis supporting Fig. 10.*) Stimulus properties are decodable at different Gabor sequence timepoints for different imaging planes. ***A***, Balanced accuracy (mean ± SEM over mice) for classifiers decoding mean Gabor patch orientations from ROI activity at each timepoint of *A-B-C-D-G* sequences (*top*) and *A-B-C-U-G* sequences (*bottom*) during session 1. Gray lines and shading show median (dark) and adjusted one-tailed 95% CIs (light), computed by shuffling orientation labels. ***B***, Same as *A* but for session 2. ***C***, Same as *A* but for session 3. In L2/3-S, stimulus orientations are decodable across much of the Gabor sequence timecourse, whereas in L5-S, they are only decodable in sessions 1 and 3, toward the end of the Gabor sequence timecourse. Similarly to L5-S, in the distal apical dendrites, stimulus orientations are generally decodable toward the end of the Gabor sequence timecourse. In particular, orientations can be decoded from more timepoints near the end of pattern-violating sequences (*U*-*G*) than the end of pattern-matching sequences (*D*-*G*). *p < 0.05 (one-tailed (upper), corrected). Statistical significance was evaluated for each timepoint. See Table 1 for details of statistical tests and the number of statistically significant timepoints in each panel.

A similar analysis was used for the timecourse orientation decoding analyses (Fig. 11). The main difference was that, in this case, decoders were trained on individual timepoints from either pattern-matching (*A-B-C-D-G*) or pattern-violating (*A-B-C-U-G*) sequences. Thus, the input data for the decoders consisted of single-frame Δ*F*/*F* responses for all ROIs together, allowing an orientation prediction to be obtained for each of the 45 timepoints in a sequence. Null CIs were obtained by training 10^4^ shuffled classifiers on each timepoint, and aggregated across mice as described above. Other than these changes, the same decoder training parameters were used as for Figure 10, including the downsampling of the pattern-matching sequence timepoint examples to match the number of pattern-violating sequence timepoint examples.

### Statistical data analysis

For most analyses, mean ± SEM is reported. In cases where the error could not be directly measured over the sample, e.g., the percentage of significant ROI USIs reported in Figure 4*D*, a bootstrapped estimate of the error was obtained by resampling the data with replacement 10^4^ times. In these cases, the SD over the bootstrapped sample is plotted instead, and this is visually signaled by the use of broader error caps (Figs. 4*D*–*E*,*H*, 9*C*,*F*).

Significance tests, unless otherwise indicated, were computed non-parametrically using permutation tests with 10^5^ shuffles to construct null distributions, based on which *p*-values could be estimated. Paired permutations were used where appropriate, namely where data could be shuffled between sessions for tracked ROIs (Figs. 4*G*,*H*, 5*B*, 7*D*,*E*), between early and late session parts, for each ROI (Fig. 3*B*) or within individual trials (Fig. 8*B*). Where *p*-values are reported, they are two-tailed, with two exceptions. First, for Figure 9, one-tailed (lower) tests were used to identify statistically significantly negative correlation values. Second, for the decoder analyses (Figs. 10, 11), one-tailed (upper) tests were used to assess whether decoding performance was statistically significantly above chance. In addition, *p*-values were Bonferroni-corrected for multiple comparisons to reduce the risk of Type I errors (false positives). Where 95% confidence intervals (CIs) are plotted, they are equivalently adjusted using a Bonferroni correction. An exception was made for Figure 8*B*, which reports the relationship between the stimuli and behavioral data. Here, Type II errors (false negatives) were considered of greater concern, and thus we reported raw two-tailed *p*-values in the panel itself. Similarly, to preserve statistical power, timecourse orientation decoder *p*-values were not corrected for the large number of timepoints decoded (45) (Fig. 11). Details of the statistical analyses for all figures, including number of comparisons and corrected *p*-values, are presented in Table 1. (The same information is also presented in Extended Table 1-1, in a spreadsheet format.)

### Analysis software

Analyses were performed in Python 3.6 (Van Rossum and Drake, 2009) with custom scripts that are freely available on GitHub (https://github.com/colleenjg/OpenScope_CA_Analysis) to be redeployed by users directly on the dataset. These scripts were developed using the following packages: NumPy (Harris et al., 2020), SciPy (Virtanen et al., 2020), Pandas (McKinney, 2010), Matplotlib (Hunter, 2007), Scikit-learn 0.21.1 (Pedregosa et al., 2011), and the AllenSDK 1.6.0 (https://github.com/AllenInstitute/AllenSDK). Dendritic segmentation was run in MATLAB 2019a (MATLAB, 2019) using the robust estimation algorithm developed by (Inan et al., 2017, 2021). Pupil tracking was performed using DeepLabCut 2.0.5 (Mathis et al., 2018). ROIs were matched across sessions using a custom-modified version of the n-way cell matching package developed by the Allen Institute (https://github.com/AllenInstitute/ophys_nway_matching).

## Results

### Responses to pattern-matching and pattern-violating stimuli evolve over days and differ between the somata and distal apical dendrites

We wanted to determine whether the responses of somata and apical dendrites to pattern-matching vs pattern-violating stimuli differ, and how those responses evolve with increasing exposure to the pattern-violating stimuli. To achieve this goal, we compared the neural responses to the two different types of stimuli over three sessions spread across multiple days. Recall that, prior to these imaging sessions, the animals had been habituated to *A-B-C-D-G* sequences with clear, repeating patterns, and that starting with the first imaging session, they were exposed to these same stimuli, but with the occasional introduction of pattern-violating *A-B-C-U-G* sequences (see Materials and Methods).

First, we examined how population-wide responses to the stimuli changed over days. In the distal apical dendritic ROIs, the difference in responses to pattern-violating (*A-B-C-U-G*) and pattern-matching (*A-B-C-D-G*) sequences increased across days, reaching statistical significance in both L2/3 and L5 by session 3 (Fig. 2*A*,*B*, *top*). In contrast, by session 3, the response differences in the somatic ROIs, which were statistically significant in session 1 for L5-S, converged towards zero (Fig. 2*A*,*B*, *bottom*). Indeed, specifically comparing the responses to the regular sequence images (*A-B-C*) and the pattern-violating images (*U-G*), we found that the average somatic ROI responses tended to decrease for both pattern-matching and pattern-violating images over time, though the effect was only statistically significant in L2/3 (Fig. 2*A*–*C*, *bottom*). In contrast, in the distal apical dendritic ROIs, we observed an increase in the average responses to the pattern-violating images, but not to the regular sequence images (Fig. 2*A*–*C*, *top*). We also examined whether some of these between-session changes could be observed within a single session (Fig. 3). We found that the difference in the responses to *D-G* and *U-G* decreased or increased significantly within individual sessions in both L2/3 and L5 distal apical dendrites, but not in the somatic planes. These results demonstrate that the responses to the pattern-violating and pattern-matching stimuli evolve differently in the somatic and apical dendritic compartments.

One potential confound that must be considered is that the *D* and the *U* images use different locations for the Gabor wavelets. As a result, different neuronal responses to these images might be driven simply by how they interact with each neuron’s receptive field. However, although this explanation might have accounted for differences in how individual neurons or dendritic segments respond to these stimuli, it is statistically highly improbable that this could account for the systematic differences observed in the population-averaged apical and somatic compartments we report here. This analysis averages over hundreds of ROIs and hence over the variations in those ROIs’ receptive field properties. Moreover, the mice’s eyes were not fixed and could move freely throughout the recording sessions, further reducing the likelihood that any particular spatial pattern differences might account for the observed ROI responses.

### The evolution of responses to pattern-matching and pattern-violating stimuli in individual somata and apical dendrite segments differ

Given our observations of changes in the responses to the Gabor sequences at the population level, we wondered whether the same effects would be observable for the tracked ROIs. This is important because changes observed in the population-wide responses could, in principle, be driven by only a few ROIs. To test this possibility, we examined the changes over days in the responses of individual ROIs. For this analysis, we made use of our USI metric (Eq. 1 in Materials and Methods) to quantify each ROI’s selectivity for the pattern-violating events. We then tracked how these USIs changed over days, for each ROI.

On day 1—when the animals were first exposed to the pattern-violating stimuli—many ROIs had clearly different responses to the pattern-matching and pattern-violating images (Fig. 4*A*, *top* and *bottom* traces). Specifically, we found that many more ROIs had strongly negative or positive USIs than would be expected by chance, as has been previously observed (Keller et al., 2012). To determine chance levels, we constructed null distributions non-parametrically for each ROI by shuffling the “pattern-matching” and “pattern-violating” labels for the stimulus images 10^4^ times, each time recomputing the USI on the shuffled data (Fig. 4*B*; see Materials and Methods). These shuffles yielded a null distribution over USI values for each ROI that reflected the null hypothesis according to which there was no difference in an ROI’s responses to pattern-matching vs pattern-violating stimuli. We then identified the percentile of each ROI’s real USI within its own null distribution: ROI USIs below the 2.5^th^ percentile or above the 97.5^th^ percentile were labeled as statistically significant (Fig. 4*B*). Across the population of ROIs, in both L2/3 and L5 somata and dendrites, there were far more significant USIs than would be predicted by chance (Fig. 4*C*,*D*). This effect was consistent across individual mice, with 10 of the 11 animals showing a statistically significant effect (Fig. 5*A*). Notably, when we restricted this analysis to sequences whose mean Gabor patch orientations occurred for both *D* and *U* images, namely 90$^\circ$ and 135$^\circ$, the USI percentages remained largely the same, meaning that USI patterns did not reflect ROI preferences for specific orientations of the Gabor patches (Fig. 4*E*). Thus, the response differences we observed were unlikely to be a result of differences in the responses to orientations of the Gabor patches in the *D* and *U* images. Together, these data reinforce our aggregate observations (Fig. 2), that the neurons respond differently to the pattern-matching vs pattern-violating stimuli.

Next, we investigated how the USIs changed over days for these tracked ROIs and found that in the somatic compartments, the USIs converged towards zero over the three sessions (Fig. 4*F*–*H*, *bottom*). In contrast, in the distal apical dendritic compartments, the USIs increased significantly over the three days. This effect was most prominent in L2/3-D (Fig. 4*F*,*G*, *top*). These effects were generally consistent across mice (Fig. 5*C*). Thus, as with the population-level data, we saw evidence for a difference in the evolution of responses in the somatic and apical dendritic compartments: Whereas somatic compartments became less sensitive to the distinction between pattern-matching and pattern-violating stimuli over sessions, apical dendritic compartments became more sensitive.

Note that, because the Gabor patch locations differ for the *D* vs *U* images, each individual neuron’s USI can be influenced by the manner in which its receptive field location overlaps with the Gabor patch locations for the *D* vs *U* stimuli, in addition to the pattern-violating vs pattern-matching nature of the stimuli. Thus, caution is warranted when interpreting individual neurons’ USI values. At the same time, the USI results are consistent with the population-wide effects (e.g., those in Figs. 2, 4*G*,*H*) which aggregate over many neurons (each with their own receptive field structure) and hence are much less affected by this potential confound.

### Somata and apical dendrites respond similarly to the onset of counter-flow

We next wondered whether the differences we observed between the somatic and dendritic compartments reflected a general difference in stimulus response properties. That is, could it be that somata simply become less sensitive to stimulus differences with exposure, whereas apical dendrites become more responsive to stimulus differences? Or, is it really something about the specifics of the pattern-matching and pattern-violation in the stimuli?

To address this question we examined the responses to a visual flow stimulus, which presented the animals a set of drifting squares, a subset of which would, at random times, start to drift in the opposite direction to the main direction of flow (counter-flow, Fig. 6*A*; see Materials and Methods). We reasoned that this stimulus should induce responses in visual cortex, but would not strongly deviate from previously experienced “patterns,” *per se*, since animals will encounter objects that move in a direction contrary to the direction of visual flow naturally in their day-to-day lives. In examining the population responses to the onset of counter-flow, we found that there was an interesting difference between layers, with L2/3 somata and dendrites responding to counter-flow onset, but not L5 somata and dendrites (Fig. 6*B*) doing so. This was further confirmed by examining the difference in the integrated Δ*F*/*F* for population responses to the onset of counter-flow and the preceding windows of time with no counter-flow. We found that the responses to the onset of counter-flow were statistically significantly larger than the responses to uniform flow in L2/3 in both somatic and dendritic compartments, and this tendency only increased slightly over sessions (Fig. 6*C*, *left*). In contrast, in L5, we found that the differences were not statistically significant and remained so across sessions (Fig. 6*C*, *right*). This is consistent with work by Jordan and Keller (2020) showing that L2/3 neurons integrate visual flow mismatch information, whereas L5 neurons do not appear to do so.

We also did not observe any apparent differences between the evolution of responses in the somatic and apical dendritic compartments in either L2/3 or L5 neurons (Fig. 6*B*,*C*). Overall, we observed fewer changes in the mean responses across sessions to the visual flow stimuli than we had observed with the Gabor stimuli (Fig. 7*A*,*B*). Similar results were obtained at the level of individual ROIs when we calculated an equivalent to the USI metric for the visual flow stimulus, with USIs that were consistent across sessions and similar across compartments (Fig. 7*C*–*E*). As such, these results do not support the hypothesis that there is a general difference in the evolution of the stimulus sensitivity of the somatic and apical dendritic compartments—either in general, or in the presence of changes in the stimulus. Rather, it suggests that the differences we observed in Figures 2 and 4 were due to the properties of our pattern-matching/violating Gabor sequences.

### Responses to pattern-matching and pattern-violating stimuli are unlikely to be driven by behavior

Given the well-documented impact of behavior on visual-neuronal activity (Niell and Stryker, 2010; Stringer et al., 2019; Salkoff et al., 2020), we wondered whether the differences in the responses to pattern-matching vs pattern-violating images could have been driven by differences in the animals’ behavior during the presentations of these two types of images. If so, these behavioral differences could be reflected in the neuronal responses in visual cortex, confounding our interpretation that the differences in neuronal response were due to the pattern-matching versus pattern-violating nature of the stimulus. To test this possibility, we compared the animals’ running velocities and pupil dilation during the pattern-matching *D* images and the pattern-violating *U* images (Fig. 8*A*). We found no significant difference in either running velocity or pupil dilation for *D* versus *U* images (Fig. 8*B*), suggesting that behavioral changes are not a major confound in our analyses. Altogether, these data support our conclusion that pattern-matching and pattern-violating stimuli are represented differently within the neocortical microcircuit. Moreover, our findings in layer 2/3 somata align with those observed in previous studies using similar stimuli (Homann et al., 2022), though we are not aware of prior work that has examined the other three compartments under similar stimulus conditions.

### Dendritic, but not somatic, ROIs reverse their selectivity for pattern-violating vs pattern-matching stimuli between days 1 and 2

Our preceding analyses showed that neurons in mouse VisP respond differently to pattern-matching and pattern-violating stimuli, that these responses evolve over days, and that there may be a difference in this evolution between compartments receiving primarily bottom-up or top-down information (Figs. 2, 4). We next sought to identify more clearly the nature of the changes in selectivity and determine whether there was a difference between the somatic and dendritic compartments.

To achieve this goal, we examined the correlation between ROI USIs in one session and the next. If an ROI’s selectivity remains largely the same over days (i.e., an ROI that prefers pattern-violating stimuli continues to prefer pattern-violating stimuli), then the second day’s USI should resemble the first day’s, plus some noise, and hence we should find positive correlations between USIs across days. Conversely, negative correlations between days are evidence of a reversal in preferred stimuli, wherein ROIs switch from responding more strongly to pattern-violating stimuli to responding more strongly to pattern-matching stimuli (or vice-versa).

To determine whether correlations were significantly different from what would be expected if there were no relationship between an ROI’s USI in one session and the next, we computed null distributions for each imaging plane and session pair by shuffling the ROI labels 10^5^ times within each session. Correlation values below these null distributions were interpreted as reflecting a statistically significant negative correlation between USI values across sessions for individual ROIs.

In the somatic compartments, we found no statistically significant correlations between ROI USIs in one session and the next (Fig. 9*A*–*C*, *bottom*). This suggests that, since the overall population tendency is for somatic ROI USIs to converge towards zero over days (Fig. 4*F*–*H*, *bottom*), individual ROI USIs on one day are not linearly predictive of their values on a subsequent day. In contrast, ROI USIs in both distal apical dendritic compartments were negatively correlated from session 1 to 2 (Fig. 9*A*–*C*, *top*). As Figure 9, *A* and *B*, shows, this reflects a tendency for the higher distal apical dendritic ROI USIs to decrease from day 1 to day 2 (*bottom right* quadrants), and for the lower ones to increase even more strongly (*top left* quadrants).

### Changes in responses to pattern-matching and pattern-violating stimuli alter stimulus decodability

So far, our analyses have demonstrated that neural activity in the somata and distal apical dendrites of L2/3 and L5 neurons in VisP reflects whether or not stimuli match previous patterns, and this property evolves across days differently in the somatic and apical dendritic compartments. We next aimed to determine whether the decodable information about the orientations of the Gabors was altered as selectivity for the pattern-matching and pattern-violating stimuli changed.

To address this question, we trained logistic regression classifiers to identify the mean Gabor patch orientation from the recorded neural responses. Using a cross-validation approach with 300 random splits, we trained the classifiers for each animal and session on 75% of the data, testing them on the remaining (held-out) 25%. Specifically, we trained the classifiers on responses to each Gabor image in order to see whether the activity associated with different images contained different amounts of orientation information. Indeed, we found that there seemed to be increasing amounts of information as the sequences progressed from *A* to *D*/*U* images. ROI responses to *A* images encoded the least amount of orientation information, with only L2/3-S having above-chance decoder performance (Fig. 10*A*). ROI responses to *B*, *C*, *D*, and *U* images each contained progressively greater numbers of sessions across planes with above-chance decoder performance levels, with *D*- and *U*-image responses each having only two sessions with chance-level decoder performance (Fig. 10*A*–*E*). L2/3-S was the only plane with responses that did not yield appreciably different decoder performance across the Gabor images, maintaining above-chance performance for all sessions and images.

Focusing on the classification of *U* images, compared to *D* images, when trained on pattern-matching *D* images, we found that both the somatic and distal apical dendrite compartment classifiers performed significantly above chance in all cases on session 1 (Fig. 10*D*). Across sessions, decoder performances remained above chance, in most cases, with no systematic upward or downward trend. However, when trained on pattern-violating *U* images, the decoding results for *U* images showed trends paralleling the evolution of the USIs in the different compartments (Fig. 10*E*). Specifically, decoding performance in the distal apical dendrites started near or at chance in session 1, clearing the chance range by session 2, at ∼60% performance. In contrast, in the somatic compartments, decoding performance started near 60%, but approached or reached chance level by sessions 2 and 3. Further analyses with a larger dataset are needed to confirm these trends. Nonetheless, these results show that both the somata and distal apical dendrites of L2/3 and L5 neurons carry detailed information about features relevant to determining whether the stimulus being viewed matches or violates the habituated pattern, and that this evolves differently over days in the somatic and apical dendritic compartments. The same trends can be seen in greater temporal detail in Figure 11. In particular, the decoder performance for the apical dendrites remains quite low during responses to images *A*, *B*, and *C*, but is generally higher for *D* and *U*. In addition, the decodability of the *U* image orientations generally increased over the sessions in the apical dendrites, but decreased in the somata, in line with the analysis run on concatenated frames (Fig. 10*E*).

## Discussion

In this study, we explored how responses to visual stimuli that either matched or violated habituated patterns change with increasing exposure. We studied this phenomenon in both somata and distal apical dendrites of pyramidal cells in primary visual cortex in order to examine potential differences between these compartments, which receive largely bottom-up and largely top-down inputs, respectively (Budd, 1998; Jiang et al., 2013; Larkum, 2013; Marques et al., 2018). First, we observed that neurons responded differently to stimuli that were pattern-matching vs pattern-violating, in line with other work (Keller et al., 2012; Homann et al., 2022). Second, we found that neural responses to the pattern-violating stimuli changed over days. In contrast, the responses to the visual flow stimuli were more stable, suggesting that the pattern-violating stimuli drove stimulus-specific changes. Third, the evolution of these responses over days differed between the distal apical dendrites versus the cell bodies. Fourth, we found that the sensitivity of distal apical dendrites to pattern-violating stimuli on one day changed systematically by the next day, but this was not observed in the somata. Finally, we found that our ability to decode orientation information about pattern-violating stimuli evolved differently in the somatic and dendritic ROIs. Importantly, for several of the analyses, we obtained the same results in both L2/3 and L5 pyramidal neurons, even though those recordings were done in different animals, which increases our confidence in the robustness of the findings.

Notably, our results do not immediately support a simple version of predictive coding wherein excitatory neurons *exclusively* encode prediction errors: this is because prediction errors associated with pattern-violating stimuli should decrease over time as the animal gains exposure to those stimuli—and can thus incorporate those stimuli into their set of experiences—whereas we see increasing sensitivity over days to the pattern-violating stimuli in the neurons distal apical dendrites. The finding that the difference in distal apical dendritic signals grows at a population level with exposure to the pattern-violating stimuli may thus be counter to proposals implementing predictive coding by using the distal apical dendrite as a site for prediction error calculations (Sacramento et al., 2018; Whittington and Bogacz, 2019). At the same time, the responses to pattern-violating stimuli in the somata did decrease over time, which is more in line with an encoding of prediction errors at the soma (but not throughout the entire cell). This suggests that different types of information are reflected in the different compartments of the neurons.

In fact, whereas the somata became less attuned to the pattern-violating stimuli over sessions, the dendritic responses became more attuned to them, as reflected by our increased ability to decode the orientations of the *U* images (Fig. 10*E*). This may reflect changes in the brain's internal model, encoded in higher-order associative regions, i.e., as an animal gains exposure to the pattern violations, they are incorporated into its higher-order models and subsequent predictions. As such, the increased decodability could reflect learning to better predict the upcoming *U* image orientations, and passing this information down through the apical dendrites. Thus, although the increased responses and decodability of the *U* images in the apical dendrites were initially surprising to us, we do not believe that they are at odds with the concept of predictive coding in visual cortex, *per se*. Rather, our observations may constrain specific predictive coding implementations and imply that models of cortical computation should treat the somata and apical dendrites differently.

Another apparent complication that our data present for a predictive coding hypothesis is that, while the Gabor stimuli evoked pattern-violation selectivity that developed over days, the visual flow stimuli did not elicit strong pattern-violation effects in the L5 somata or apical dendrites on any of the recording days. Moreover, for both somata and apical dendrites, the L2/3 ROIs were sensitive to the differences between the *D* and *U* images on session 1 and remained so over the three sessions. Why would this visual flow stimulus produce different evolution over sessions as compared to the Gabor sequences? One possible explanation is that the onset of the counter-flow is itself never predictable (the timing of that onset was randomized in our experiments), and, at the same time, the animals have likely encountered objects moving counter to the direction of visual flow during their everyday activities. Hence, these stimuli may trigger a different form of prediction error, i.e., for a commonly-encountered stimulus property occurring at an unpredictable time. This is very different from our Gabor stimulus, where the timing of the pattern violation is more predictable, but the content of the pattern violation is initially unpredictable. Our results thus imply that different forms of pattern-violating stimuli may elicit different forms of prediction error in the neocortical microcircuit. Future work should further explore responses to different forms of pattern-violating stimuli.

There are a number of limitations to this work that must be recognized. First, as noted, we were not recording somata and distal apical dendrites in the same neurons. Thus, even though we saw very different evolutions in the responses of the distal apical dendrites and somata to the Gabor sequence stimulus, we cannot say with certainty that these differences hold within individual cells. Previous studies that have examined coupling between somata and distal apical dendrites within individual recording sessions have shown mixed results. Distal apical L2/3 dendrites in mouse VisP have been shown to have local bursting events that have similar but qualitatively different orientation selectivities compared to those from their simultaneously recorded somata (Smith et al., 2013). Other studies have found strong coupling between dendrites and somata (Beaulieu-Laroche et al., 2019; Francioni et al., 2019). However, those studies recorded from locations in L5 pyramidal neurons that were much closer together than in the present work, and data from mouse motor cortex demonstrates that the coupling strengths fall off exponentially with distance for L2/3 and L5 pyramidal neurons (Kerlin et al., 2019). Moreover, this last study found that task-associated events were clustered and compartmentalized within ∼10 μm inside dendritic-branch segments and not widely spread throughout the dendritic tree. Research in rat cortex has shown that inhibition can modulate the coupling of somata and distal apical dendrites of L2/3 pyramidal cells, providing a potential mechanism for context-dependent coupling (Larkum et al., 2007).

Together, this body of work supports the idea that the strength of the coupling between somata and distal apical dendrites in L2/3 and L5 neurons varies depending on context. Consistent with this potential context dependence, we saw clear differences in the evolution of selectivity for pattern-violating Gabor sequences over time between the somatic and distal apical dendritic compartments, but we did not see these differences in response to the pattern-matching Gabor sequence images (Figs. 2*C*, 5*B*) or the visual flow stimuli (Fig. 7). Since these observations were reasonably consistent across mice (Fig. 5*C*), it seems likely that these results would hold within individual neurons. At the same time, future work using simultaneous multiplane imaging will be critical to confirm and expand upon these findings. In summary, our findings of differences between somata and distal apical dendrites are consistent with previous reports of imperfect and potentially context-dependent dendro-somatic coupling in mouse cortical pyramidal cells.

A second relevant limitation is that, though we examined the distal apical dendrites separately from the somata specifically in order to identify potential differences in the processing of top-down and bottom-up inputs, an ideal experiment would record simultaneously from other higher-order brain regions and their projections into visual cortex (Leinweber et al., 2017; Marques et al., 2018). This would help determine whether the signals we saw in the distal apical dendrites were being calculated locally or in other regions.

A third limitation, given the nature of our visual stimuli, is that we were unable to measure either the classical receptive fields or the orientation tuning of the neurons. As such, we cannot state with certainty whether these factors could explain the differences in how individual cells responded to pattern-matching vs pattern-violating stimuli. However, we observed our results in aggregate across large populations of recorded neurons, with likely diverse orientation tuning properties and receptive fields. Moreover, the mice’s eyes could and did move continuously throughout the sessions, so that the same places on the screen fell onto different neurons’ receptive fields at different times. Thus, it is unlikely that idiosyncracies of individual neurons’ orientation selectivities or receptive field locations could entirely account for the pattern-violating event responses. This assertion is supported, in part, by our finding that even when we only compared responses for pattern-matching and pattern-violating images with the same mean orientation we still observed significant differences in the responses (Fig. 4*E*). Moreover, we observed significant changes in pattern-violating event selectivity over days, whereas classical receptive fields and orientation tuning of neurons in mouse VisP are known to be relatively stable over these timescales (Montijn et al., 2016).

Fourth, these experiments were open-loop, and thus did not incorporate any of the patterns of sensorimotor coupling that the animal would have experienced for a lifetime. On one hand, this is a limitation as it reduces our ability to directly compare our results to the numerous reports of apparent sensorimotor predictions and prediction error signals in visual cortex (Keller et al., 2012; Zmarz and Keller, 2016; Leinweber et al., 2017). On the other hand, the fact that we saw evidence for an evolution of responses in the open-loop setting suggests that these changes might be driven by pattern violations in sensory data, even in the absence of sensorimotor feedback. Notably, the open-loop nature of our experiments also makes our experimental paradigm different from the situation modeled by Sacramento et al. (2018) and Whittington and Bogacz (2019), wherein top-down supervisory signals were assumed to be present. This could be another reason why we observed prediction-error like activity in the somata and not the apical dendrites.

Finally, it must be recognized that different sensory stimuli, which can present different forms of pattern violation, and recordings in different brain regions may produce different results. To more fully assess hierarchical predictive processing, future work should thoroughly explore the space of possible pattern-matching and pattern-violating sensory stimuli, and recordings of other regions of the neocortex.

A long-standing goal of neuroscience is to understand how our brains process the sensory data that we receive from the world around us. In this work, we monitored changes in the responses of visual cortical neurons in mice that were exposed to external stimuli violating habituated patterns, and found that these changes were different in the somatic and distal apical dendritic compartments of both supra-granular and sub-granular pyramidal neurons. Looking forward, we anticipate that these findings could help uncover models describing the brain’s hierarchical predictive processing and mechanisms of learning.
